# Prevalence of microalbuminuria in hypertensive patients and its associated cardiovascular risk in clinical cardiology: Moroccan results of the global i-SEAR CH survey – a sub-analysis of a survey with 21 050 patients in 26 countries worldwide

**DOI:** 10.5830/CVJA-2010-021

**Published:** 2010-08

**Authors:** R Habbal, AR Sekhri, M Volpe, i-Search Investigators

**Affiliations:** Cardiology Department, Ibn Rochd University Hospital, Casablanca, Morocco; Medical Affairs Department, sanofi-aventis, Casablanca, Morocco; University of Roma ‘La Sapienza’, 2nd Faculty of Medicine, S Andrea Hospital, Rome and IR CCS, Neuromed, Italy; University of Roma ‘La Sapienza’, 2nd Faculty of Medicine, S Andrea Hospital, Rome and IR CCS, Neuromed, Italy

**Keywords:** microalbuminuria, prevalence, cardiology, hypertension, irbesartan, risk factors

## Abstract

**Objectives:**

To determine the prevalence of microalbuminuria (MAU) in hypertensive outpatients visiting a cardiologist’s office or clinic and to describe the relationship between MAU and cardiovascular risk factors.

**Methods:**

This was an international, observational, cross-sectional study of 22 282 patients, with 457 subjects from Morocco in 40 cardiology centres. Inclusion criteria were: male and female outpatients aged ≥ 18 years with currently treated or newly diagnosed hypertension (≥ 140/90 mmHg at rest on the day of the study visit) and no reason for false positive microalbuminuria dipstick tests.

**Outcome measures:**

Prevalence of microalbuminuria assessed using a dipstick test, co-morbid cardiovascular risk factors or disease and their relationship with the presence of MAU, and role of pharmacotherapy in modulating the prevalence of MAU.

**Results:**

The prevalence of microalbuminuria in hypertensive patients in Morocco (67.8%) was high compared to the worldwide prevalence (58.3%). Despite the fact that all physicians regarded MAU as important for risk assessment and therapeutic decisions, routine MAU measurement was performed in only 35% of the practices. In clinical cardiology, MAU is highly correlated with a wide variety of cardiovascular risk factors and cardiovascular disease.

While angiotensin receptor blockers (ARBs) appeared to be associated with the lowest risk of MAU, calcium channel blockers (CCBs) were more often used in this patient group.

**Conclusions:**

Hypertensive, high-risk cardiovascular patients are common in clinical cardiology. Given the high prevalence detected, screening of MAU in addition to more aggressive multi-factorial treatment to reduce blood pressure as well as other cardiovascular risk factors is required.

## Summary

The presence of albumin in the urine indicates a disturbance in the barrier function of the endothelial glomerular cells (podocytes).^1^,^2^ This parameter is measured in mg per 24 hours or μg/ml urine. Excretion of albumin into the urine must be detected as early as possible, because once pathological thresholds are reached, progression to advanced renal disease may result. The range of 30–300 mg/24 hours, termed microalbuminuria (MAU), is accompanied by an increased incidence of clinical proteinuria, an increase in serum creatinine, and more frequent development of terminal renal insufficiency, in addition to increased cardiovascular risk.^3^

Albuminuria is frequently regarded as a disease of the kidneys. However, there is often a simultaneous secretion of albumin into the retinal bed (cotton wool spots).^4^ Moreover, pre-clinical studies have shown that, using labelled albumin, this disorder is also present in the whole vascular system including the myocardium^5^ and brain.^6^-^8^

From a cardiologist’s standpoint, therefore, albuminuria is of critical importance to determine the prognosis of patients with cardiovascular disease. Patients with myocardial infarction, for example, have a worse prognosis if albuminuria is present compared to no albuminuria.^9^-^11^ Furthermore, in patients with normal coronary arteries on angiography, the extent of endothelial dysfunction has been shown to correlate with the degree of albumin excretion.^12^ Overall it has been shown that MAU is more important than many established cardiovascular risk factors for the prediction of the further course and outcome.^13^

The International Survey Evaluating microAlbuminuria Routinely by Cardiologists in patients with Hypertension (i-SEARCH) was undertaken in 26 countries around the world in a total of 1 750 sites to provide epidemiological data on the prevalence of MAU and its associations with established cardiovascular risk markers and disease. This article reports the data for Morocco, worldwide results having been published recently.^14^

## Methods

This was an international, observational, cross-sectional study in which subjects were evaluated during a single visit (methods have been published previously^14^). It had a two-step epidemiological design. In the first step, prior to patient recruitment, participating physicians completed a site questionnaire that documented practice location (urban, suburban or rural) and type (community or hospital based) as well as duration of service, degree of awareness and experience in detection of MAU, and its clinical relevance. In the second step, at each site, consecutive patients fulfilling eligibility criteria were invited to participate in the study. The study was conducted in accordance with the ethical principles of the current Declaration of Helsinki and consistent with the international conference on harmonisation and good clinical practice.

Inclusion criteria were as follows: male and female outpatients aged 18 years or older, with currently treated or newly diagnosed arterial hypertension, defined as a seated systolic (SBP)/diastolic blood pressure (DBP) of ≥ 140/90 mmHg at rest on the day of the study visit. Patients with acute fever (> 38°C), renal disease (serum creatinine > 20 mg/l), concomitant urinary tract infection, treated with cimetidine, or having undertaken strenuous physical activity in the preceding 24 hours, as well as female subjects who were pregnant or menstruating were ineligible to participate due to the likelihood of false positive results.

Once enrolled, demographic data, cardiovascular history, risk factors and co-morbidities, symptoms and signs of cardiovascular disease and current chronic drug therapy were documented on the case report form. The following measurements were then carried out on each patient: heart rate, urinary albumin and creatinine concentration, and waist and hip circumference. To ensure consistency between study sites, all centres performed dipstick screening for MAU with sponsor-provided reagent strips (Microalbustix®), which have a sensitivity of 82.6%,^15^ and followed a standardised sample-collection and testing procedure. Urine albumine levels were grouped into categories: 10, 30, 80 or 150 mg/l.

The primary objective of this study was to define the prevalence of MAU in hypertensive outpatients attending a cardiologist. Secondary objectives were to establish a correlation between the prevalence of MAU and known cardiovascular risk factors in the study population, and to increase physicians’ awareness of the importance of MAU screening to identify at-risk patients.

## Statistical analyses

Population characteristics were recorded as numbers, means and standard deviations, together with 95% confidence intervals (CI) for the means of quantitative variables, and numbers and percentages with 95% CI of the population for categorical data. Outcomes included prevalence of MAU with 95% CI, taking into account the cluster design effect using the Proc SURVEYMEANS in SAS for categorical variables. The association between high levels of MAU and cardiovascular risk factors was studied and odds ratios were calculated. For the calculations SAS version 8.2 was used.^16^

## Results

Over the six months’ recruitment period, 476 patients were screened at 40 cardiology practices in Morocco (22 282 worldwide). One patient did not sign the informed consent and 18 patients did not meet the pre-specified inclusion and exclusion criteria or had no documented albumin or creatinine values. The primary analysis was done on 457 patients.

Patients were on average 59.1 ± 11.1 years old, and 59.7% were female. The majority (81.4%) had uncontrolled hypertension (SBP ≥ 140 mmHg and/or DBP ≥ 90 mmHg) of duration 4.5 ± 4.5 years, and the mean blood pressure was 161.4 ± 27.7/91.1 ± 14.1 mmHg on the day of the study. Cardiovascular risk profiles of the Moroccan patients compared to the worldwide study population are summarised in Table 1.

**Table 1.** Cardiovascular Risk Profile (Primary Analysis Population)

*Parameter (mean ± SD or %)*	*Morocco (n = 457)*	*Global survey (n = 21 050)*
Demographics		
Age (years)	59.1 ± 11.1	62.4 ± 11.7
Male gender (%)	40.3	52.3
BMI (kg/m^2^)	28.6 ± 5.8	28.9 ± 5.7
Hypertension		
Duration (years)	4.6 ± 4.5	8.1 ± 7.7
SBP (mmHg)	161.4 ± 27.	149.2 ± 20.2
DBP (mmHg)	91.1 ± 14.1	87.4 ± 11.8
Proportion uncontrolled (140/90 mmHg) (%)	81.4	76.8
Heart rate/sinus rhythm		
Heart rate (bpm)	74.7 ± 12.3	74 ± 12
Sinus rhythm: yes (%)	96.4	94.8
Cardiovascular risk factors		
Family history MI/CAD (%)	14. 4	27.8
Regular physical activity (%)	17.7	35.0
Current/former smoker (%)	6.8/5.3	14.2/20.5
Current diabetics (%)	19.1	27.5
Diabetes type 1/type 2 (%)	11.5/88.5	4.9/95.1
Duration of diabetes (years)	6.8 ± 5.5	7.9 ± 7.7
Additional risk factors		
Total cholesterol (mmol/l)	5.5 ± 1.2	5.3 ± 1.1
HDL-C (mmol/l)	1.3 ± 0.5	1.3 ± 0.5
LDL-C (mmol/l)	3.0 ± 1.4	3.2 ± 1.0
Triglycerides (mmol/l)	1.6 ± 1.0	1.8 ± 1.0
CRP (mg/dl)	1.1 ± 0.9	0.9 ± 0.9
Serum creatinine (mmol/l)	91.9 ± 26.9	89.9 ± 23.8
Creatinine clearance (ml/min)	90.0 ± 35.5	87.9 ± 34.1
< 30 ml/min (%)	0	0.7
30–60 ml/min (%)	21.3	19.3
60–80 ml/min (%)	23.7	26.5
80–120 ml/min (%)	36	38.8
> 120 ml/min (%)	19	14.8
Co-morbidities		
Coronary artery disease (%)	10.1	22.9
Congestive heart failure (%)	4.4	5.8
Atrial fibrillation (%)	4.2	8.3
History of ischaemic stroke	8.6	4.8
History of TIA (%)	4.4	3.8
Peripheral artery disease (%)	3.1	4.2
Other cardiovascular disease		
LVH (indice de Sokolow en mm)	31.7 ± 9.4 (*n* = 246)	24.8 ± 9.8 (*n* = 8 311)
Ejection fraction ≤ 40% (%)	4.9	4.7
Carotid stenosis (%)	1.3	2.9
Aortic aneurysm (%)	0.4	1.4

SD: standard deviation, LVH: left ventricular hypertrophy, TIA: transient ischaemic attack, CRP: C-reactive protein, MI: myocardial infarction.

**Fig. 1.**
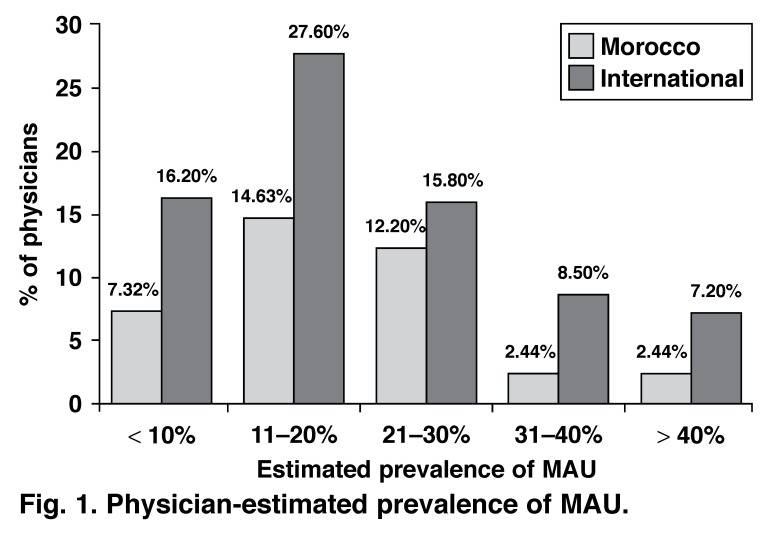
Physician-estimated prevalence of MAU .

According to Moroccan physicians, MAU is routinely measured in 35% (95% CI: 22–51%) of their practices versus 37% (95% CI: 35–40%) globally. When asked to estimate the frequency of MAU in hypertensive patients, 14.6% of physicians estimated it between 11 and 20% (27.6% globally). However, 61% of the physicians did not provide an estimation of prevalence of MAU (Fig. 1).

On the other hand, presence of MAU seemed to influence 97.4% of treatment decisions and particularly those relating to treatment of blood pressure (100%). Furthermore, 86.1% of physicians said that MAU also influenced decisions related to achieving glycaemic control. While the majority (95.12%) of physicians linked presence of MAU to a patient’s prognosis, all of them (100%) also felt that a diagnosis of MAU was relevant to improving the management of other cardiovascular risk factors. These attitudes were largely comparable with their colleagues worldwide.

**Fig. 2.**
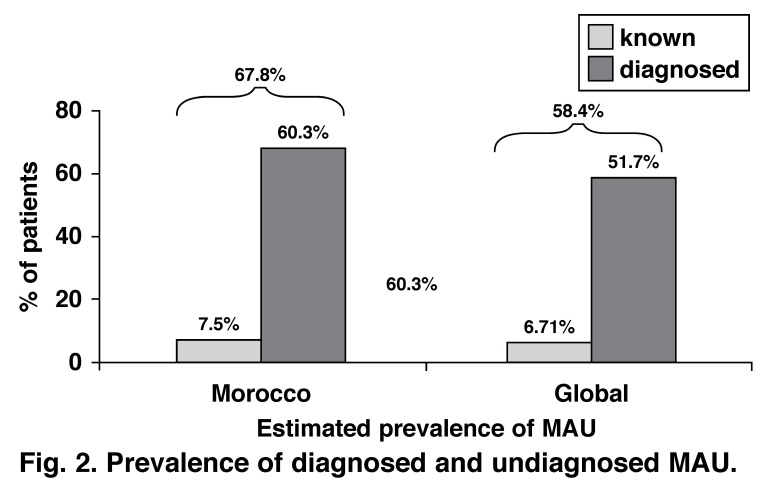
Prevalence of diagnosed and undiagnosed MAU .

Within the primary analysis of the Moroccan population, few patients had impaired renal function and 7.5% had previously known albuminuria. However, urinalysis with a once-off dipstick test revealed that 67.8% of the study population had evidence of MAU (58.4%, globally), with prevalence rates slightly higher in men (69.0%) than women (67.0%) (Fig. 2).

Correlations between several cardiovascular risk factors and MAU were tested in the global study. The following factors appeared to be associated with presence of MAU: male gender, large waist circumference, SBP ≥ 120 mmHg, DBP ≥ 100 mmHg, creatinine clearance ≥ 50 ml/min, and the presence of diabetes, congestive heart failure, coronary artery disease (CAD), history of cerebral pathology, peripheral artery disease (PAD), dyspnoea or palpitations. On the other hand, MAU occurred less often in patients who had regular physical activity (< 4 h/week).

**Fig. 3.**
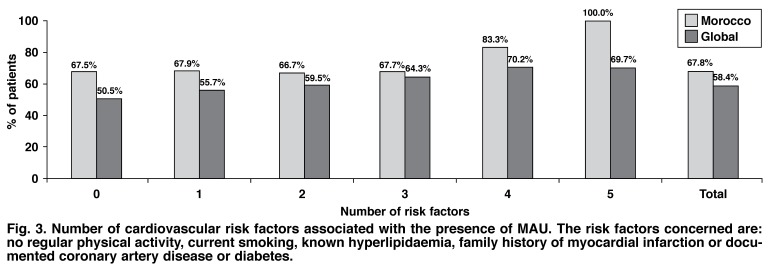
Number of cardiovascular risk factors associated with the presence of MAU . The risk factors concerned are: no regular physical activity, current smoking, known hyperlipidaemia, family history of myocardial infarction or documented coronary artery disease or diabetes.

**Fig. 4.**
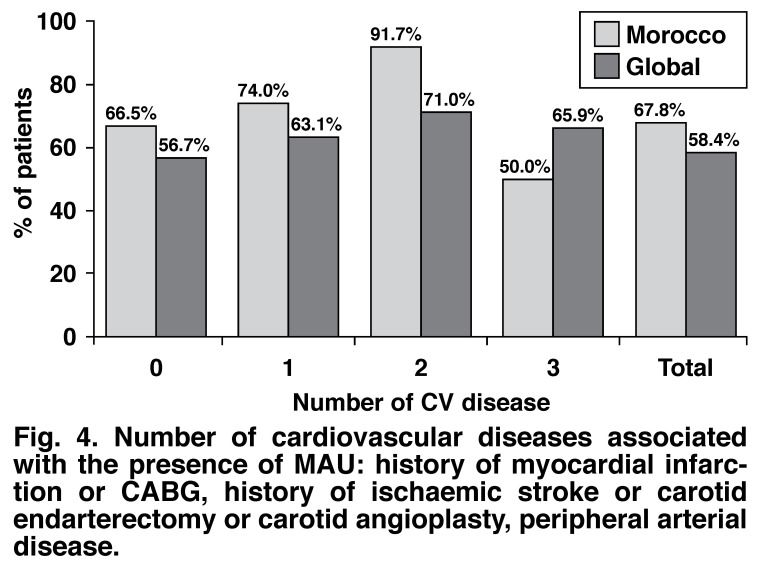
Number of cardiovascular diseases associated with the presence of MAU : history of myocardial infarction or CABG, history of ischaemic stroke or carotid endarterectomy or carotid angioplasty, peripheral arterial disease.

Table 2 summarises the prevalence of MAU when associated with these parameters in the Moroccan population in this study. The number of cardiovascular risk factors and cardiovascular diseases associated with the presence of MAU are shown in Figs 3 and 4.

**Table 2.** Prevalence Of MAU When Associated With These Parameters

*Risk factors*	*Number of patients*	*MAU prevalence (%)*
Gender:		
male	184	69.02 (61.0–75.3)
female	273	67.03 (61.3–72.3)
Waist circumference:		
high	290	72.41 (67.0–77.2)
normal	160	59.38 (51.6–66.7)
Blood pressure: (mmHg)		
SBP: ≥ 180	138	72.46 (64.5–79.2)
120–129	38	65.79 (49.9–78.8)
DBP: ≥ 110	49	71.43 (57.6–82.2)
80–84	113	69.03 (60.0–76.8)
Pulse pressure: (mmHg)		
> 80	100	66 (56.3–74.5)
51–60	108	63.89 (54.5–72.3)
Triglycerides:		
high (≥ 1.69 mmol/l)	73	73.97 (62.9–82.7)
low (< 1.69 mmol/l)	101	61.39 (51.6–70.3)
Diabetes:		
diabetes (+)	87	68.97 (58.6–77.7)
diabetes (–)	368	67.39 (62.4–72.0)
Regular physical activity:		
yes	81	62.96 (52.1–72.7)
no	376	68.88 (64.0–73.4)

Use of antihypertensive pharmacotherapy was more frequent in patients with the presence of MAU. Therefore CCBs (29 vs 21%), ACE inhibitors (32 vs 27%), thiazide diuretics (37 vs 31%) and beta-blockers (43 vs 42%) were more frequently prescribed in patients with MAU compared to patients without MAU. However patients with MAU received ARBs less often (23%) compared to patients without (25%).

**Fig. 5.**
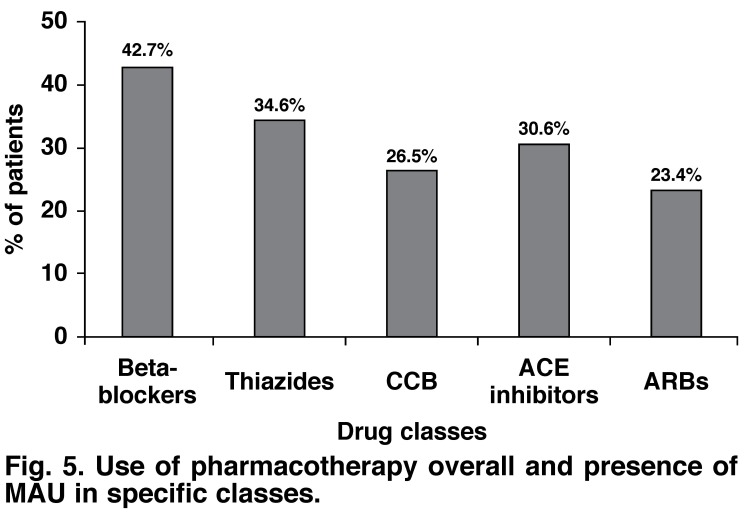
Use of pharmacotherapy overall and presence of MAU in specific classes.

Overall, the pharmacotherapy prescribed to patients with MAU was in the following order: beta-blocker > thiazide diuretics > ACE inhibitors > CCBs > ARBs (Fig. 5). The difference between prescriptions for CCBs and ARBs was significant.

## Discussion

The demographic characteristics of the present sample indicated that hypertensive high-risk patients are common in clinical cardiology. The population was mostly elderly with a substantial cardiovascular risk-factor profile and a considerable burden of co-morbidity. Therefore, microalbuminuria is not only a risk marker for diabetic nephropathy but also indicates a considerable increase in cardiovascular risk. The investigation of the interdependence of cardiovascular risk and microalbuminuria was of particular value in this patient population.

The present sub-analysis of the Moroccan centres of the global i-SEARCH survey generated the following key results: (1) patients in Morocco were grossly comparable to the patient population of the global survey, except for a lower rate of diabetes and a higher rate of uncontrolled blood pressure; (2) the prevalence of MAU in hypertensive patients in clinical cardiology (67.83%) exceeded that found in the general population and in primary care, while physicians consider MAU as a cardiovascular risk marker; (3) MAU was associated with a number of cardiovascular risk factors and disease; (4) beta-blockers were more frequently prescribed in MAU-positive patients compared to ARBs or ACE inhibitors.

The prevalence of MAU found in this sample of hypertensive patients in a cardiological outpatient setting indicates that this cardiovascular risk factor is very common in clinical cardiology (67.83%). It was furthermore, substantially higher than found in studies on unselected persons in the general population,^17^,^18^ and patients in primary care.^19^,^20^ The HYDRA study in primary care, for example,^19^,^21^ has documented a prevalence of 21.2% of patients with hypertension and 37.8% of patients with both hypertension and diabetes. The global DEMAND study has documented a prevalence of MAU and hypertension of 39% in general practice.^20^

Explanations for the higher prevalence of MAU in i-SEARCH may be as follows: the study population was older than in most previous studies, and 27.5% of the enrolled hypertensive patients were diabetic, whereas in other studies,^22^-^23^ diabetic subjects were excluded. Patients with known albuminuria were also not excluded as was the case in the DEMAND study.^20^ This and the high-risk cardiovascular population attending a cardiologist in comparison to those seeking primary care may account for the observed differences and the higher prevalence reported in the present study.

Comparing the Moroccan results to the global ones, it is apparent that the prevalence of MAU is higher in Morocco (67.83 vs 58.4%). Despite this, the estimated true prevalence by physicians in their patient cohort, its assessment and the use of this marker for therapeutic decisions is low. This finding has also been documented for general practitioners (HYDRA).^19^ It reflects a discrepancy between physician awareness of the prognostic importance of MAU and actual screening for MAU in cardiology practice.

MAU was associated with a number of cardiovascular risk factors and disease in the present study. This observation is in line with previous data stemming from population-based studies^17^ and primary care.^19^,^21^ It indicates that MAU is common in patients referring to cardiology departments and is associated with a number of other cardiovascular risk factors. This association was previously described in clinical studies for males^24^ and older patients,^25^ those with diabetes,^26^ obesity,^27^ smoking,^28^ insulin resistance syndrome,^29^ left ventricular hypertrophy (LVH),^30^ left ventricular dysfunction^31^ and C-reactive protein^32^ (not significant in the present study).

While not all parameters could be confirmed in the present study, the strong association of MAU with a variety of cardiovascular risk markers was evident. For instance, the prevalence of MAU in both diabetic and non-diabetic patients within the Moroccan population appeared to be comparable, whereas diabetes has been established as an important risk for MAU (OR: 1.24; 1.12–1.38) in the larger population of the global study.^14^

Interestingly, the prevalence of MAU was on the other hand particularly low in patients with more than four hours per week of regular exercise or those with high HDL cholesterol levels. This finding is in line with previous reports that microalbuminuria is low in physically active patients and can even be reversed when patients are motivated to exercise.^33^

## Therapeutic implications

A wide spectrum of treatment including statins, ACE inhibitors and ARBs has been shown to improve endothelial dysfunction, microalbuminuria and proteinuria. In the IDNT study,^34^ for example, the ARB irbesartan has been shown in patients with hypertension, diabetes and nephropathy to prevent the further deterioration of proteinuria in comparison to the CCB (amlodipine). In the IRMA-2 study in patients with hypertension, diabetes and microalbuminuria, it was even shown that early intervention resulted in a reversal and normalisation of albumin excretion.^35^

Evidence favouring ARBs over beta-blockers comes from a sub-analysis of the LIFE trial. Ibsen and colleagues compared atenolol and losartan with regard to the cardiovascular outcomes in patients with MAU and showed that a reduction in MAU was associated with a significantly reduced risk of non-fatal myocardial infarction, stroke and cardiovascular death.^36^

Therefore, it was of particular interest to test the differences between antihypertensive classes with regard to MAU in clinical practice. The interpretation of analyses was difficult because of unknown variables and the cross-sectional nature of the study, but it revealed that beta-blockers and CCBs were more widely prescribed than the ARBs in MAU-positive patients. It is possible to at least assert that the choice of antihypertensive drug was not in line with the study results discussed above.

Microalbuminuria is also a justified target for primary prevention, as seen in evidence of recent compelling results from the PREVENT-IT study.^37^ Healthy individuals with microalbuminuria, but without hypertension or hypercholesterolaemia, were treated either with placebo or RAS blockade. At four years’ follow up, microalbuminuria was effectively reduced, which was associated with a 44% reduction in cardiovascular events.

## Strength and limitations

The main strengths of our cross-sectional study included a large, referred cohort of hypertensive patients attending a cardiologist or internist, with validation of predefined primary and secondary endpoints. However, two limitations should be noted. First, microalbuminuria could only be assessed on a single occasion although guidelines recommend triple testing (two out of three tests need to be positive). Therefore the present data may not allow an exact quantification of how many patients would be positive or negative on a second occasion. However, other data suggest that this requirement will reduce the point prevalence by only one-fifth,^38^ up to a maximum of one-third.^39^ Second, a follow up would allow a closer investigation of the relationship between ARB use and the development or regression of microalbuminuria.

## Conclusions

A high prevalence of microalbuminuria was detected in a random sample of hypertensive patients attending a cardiology outpatient setting, indicating that high cardiovascular risk is common in clinical practice. Early detection, in addition to a more aggressive multifactorial treatment based on inhibitors of the renin–angiotensin system (RAS blockade) to reduce blood pressure as well as other cardiovascular risk factors is warranted to facilitate not only secondary but also primary prevention.
